# Cardio-oncology Clinical Assessment and Screening in Patients Undergoing High Toxicity Chemotherapy: A Retrospective Cohort Study

**DOI:** 10.7759/cureus.32513

**Published:** 2022-12-14

**Authors:** Carlos A Regino, Jonathan Cardona-Vélez, Jesus D Bello Simanca, Andres F Miranda Arboleda, Juan G Gamboa Arroyave, Fabian Jaimes

**Affiliations:** 1 Internal Medicine, Hospital Alma Mater de Antioquia, Medellín, COL; 2 Cardiology, Bolivarian Pontifical University, Medellín, COL; 3 Faculty of Medicine, University of Antioquia, Medellin, COL; 4 Emergency Department, Hospital Pedro Leon Alvarez Diaz, La Mesa, COL; 5 Division of Cardiology, Kingston Health Science Centre, Kingston, CAN; 6 Cardiology, Hospital Pablo Tobón Uribe, Medellin, COL; 7 Internal Medicine, Hospital Pablo Tobon Uribe, Medellin, COL; 8 Internal Medicine, Universidad de Antioquia, Medellín, COL

**Keywords:** cardio-oncology, solid tumors, hematological malignancies, chemotherapy, cardiotoxicity

## Abstract

Objective: To describe the clinical characteristics and cardio-oncological assessment in patients undertaking highly toxic chemotherapy and/or chest radiotherapy in a high-complexity hospital.

Methods: A single-center retrospective cohort study was carried out between January 1st, 2017 and December 31st, 2019. The medical records of patients with solid or hematological neoplasms were reviewed. Descriptive information was obtained on demographic characteristics, chemotherapeutic agents, pre-chemotherapy cardiovascular (CV) evaluation, and CV outcomes. The risk of complications was assessed using the Mayo Clinic risk score.

Results: A total of 499 patients were included, the most common neoplasm was non-Hodgkin's lymphoma (21.6%), followed by breast cancer (19.4%). A very high risk of cardiotoxicity was present in 44.1% and 90% were not evaluated by cardiology. Pre-chemotherapy echocardiography was obtained in 65%, but only 19.4% underwent echocardiographic control after finishing chemotherapy. The most frequent CV outcomes were chemotherapy-related systolic dysfunction (4.4%) and rhythm disturbances (2.8%), with atrial fibrillation and atrial flutter being the most frequent arrhythmias.

Conclusion: Despite the recognized CV toxicity of chemotherapeutic drugs, the majority of patients receiving highly toxic regimens at high risk of CV complications are not previously evaluated by a cardiologist and the CV workup was not routinely used in our study. The implementation of cardio-oncology programs will facilitate the identification of high-risk patients, aiming to detect and treat complications early.

## Introduction

An estimated 19.3 million new cancer cases and almost 10 million deaths occurred in 2020 worldwide. Female breast cancer has surpassed lung cancer as the most frequent neoplasm, with an estimated 2.3 million new cases (11.7%), followed by lung (11.4%), colorectal (10.0 %), prostate (7.3%), and stomach (5.6%) [1]. It is estimated that 1,918,030 new cases and 609,360 cancer deaths are projected to occur in the United States in 2022 [2].

In recent years, an important evolution has been observed in the treatment of this condition, with new therapies that have led to a decrease in mortality [2-4]. For example, breast cancer death rates decreased by 34% from 1990 to 2010 [5], with similar numbers in malignancies of hematological origin [6].

Paradoxically, the benefits obtained with the new treatments have been accompanied by an increase in morbidity, with cardiovascular disease (CVD) being the second cause of morbidity and the main cause of death in cancer survivors [7-8]. This complication can occur acutely or chronically, and even several years after completion of therapy [7, 9].

Cardiotoxicity refers to the adverse CV effects produced by cancer therapies, which include an increased risk of decreased ventricular function, arterial hypertension, coronary artery disease, valvular heart disease, thromboembolic events, pericarditis, and arrhythmias [9-10]. Due to the impact of potential CV disease, it is essential to find effective interventions for its prevention and treatment, as the timely evaluation and follow-up of the patients before, during, and after chemotherapy [11-13].

In Latin America, few studies describe the clinical and sociodemographic characteristics of patients at risk of developing CV manifestations associated with cancer therapy. In highly toxic chemotherapy specifically, the racial and socio-cultural characteristics of our population could lead to a different impact and presentation of CV toxicity in cancer patients.

The objective of this study was to determine the clinical characteristics and the cardio-oncological evaluation of patients undergoing chemotherapy and their respective CV outcomes.

## Materials and methods

Study design

A retrospective cohort study.

Scenario

It was carried out in a high-complexity teaching hospital in the city of Medellín, from January 1, 2017, to December 31, 2019.

Participants

Patients older than 18 years with a diagnosis of solid or hematological neoplasms, who received highly toxic chemotherapy (defined as the combination of two or more antineoplastic agents in which it is expected, according to the approval study, Grade III toxicity ≧10% and/or Grade IV ≧5%) and/or chest radiotherapy) [14]. Patients with incomplete information about chemotherapy and CV outcomes or who received treatment with palliative intention were excluded. 

Variables

Sociodemographic variables, clinical past history, types of neoplasms, treatments, and CV laboratory tests were recorded. The risk of cardiotoxicity was classified according to the Mayo Clinic scoring system as very low (0), low (1-2), intermediate (3-4), high (5-6), and very high (>6) risk. This classification system is a multimodal system and it is the result of the risk factors of each patient and the score assigned to the risk categories related to chemotherapy, as shown below [15].

Patient risk factors

We used the Mayo Clinic risk score that attributes one point to every one of these factors: heart disease or heart failure, coronary artery disease or equivalent, arterial hypertension, diabetes mellitus, previous or current use of anthracyclines, previous or current chest radiation, female gender, age 65 years. Each one of these variables: chemotherapy-related risk scored based on the known potential CV involvement of every drug category: High (4 points): anthracyclics, trastuzumab, cyclophosphamide, ifosfamide, clofarabine. Moderate (2 points): docetaxel, pertuzumab, sorafenib, sunitinib. Low (1 point): imatinib, lapatinib, bevacizumab, dasatinib. Rare (0 points): rituximab, etoposide, thalidomide.

Outcomes

The CV outcomes potentially related to the previously mentioned risk categories included: post-chemotherapy left ventricular systolic dysfunction (LVEF 10% and/or decreased global longitudinal strain >15%), stroke, nonfatal acute myocardial infarction, hospital admission for CV causes, arrhythmias, and all-cause mortality.

Information resources

The information was obtained from secondary sources such as the institution's electronic medical record and the results of laboratory tests and CV work-up carried out during follow-up. The study was approved by the Institutional Research Ethics Committee. The patient’s identity was protected and due to the observational nature of this study, no experimental intervention was performed that could represent a risk for study participants.

Bias

Selection bias was controlled from the registry of patients who met the inclusion criteria. Information bias was controlled by training the research crew in data collection.

Sample size

Non-probabilistic convenience sampling was performed, including all patients who met the inclusion criteria.

Statistical methods

A database was designed where the information obtained was systematically recorded. Qualitative variables were described by absolute and relative frequencies; the quantitative data were analyzed as mean and standard deviation or median and interquartile range, depending on their distribution. The information was analyzed using the statistical package SPSS (SPSS 24 Inc, Chicago, IL).

## Results

Participants

**Figure 1:**
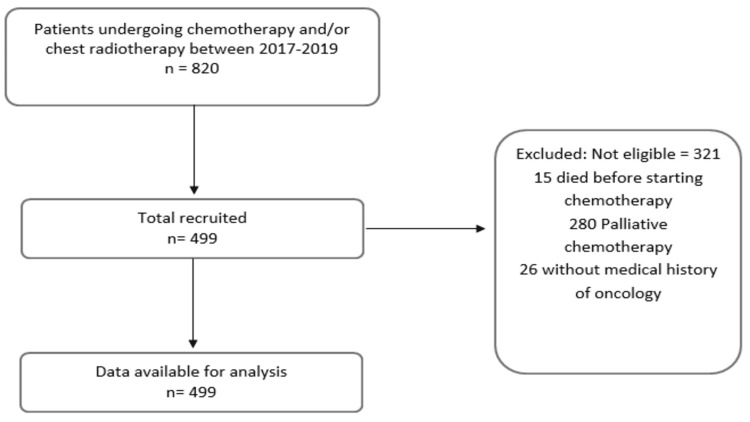
Recruitment of 499 patients with a diagnosis of solid or hematological neoplasia undergoing chemotherapy and/or chest radiotherapy.

We screened 820 potential patients. After applying inclusion and exclusion criteria, 321 patients were excluded and we finally got a cohort of 499 patients (Figure 1).

Descriptive data

The median age was 58 years, 58% were women (n=289), and the main comorbidities were arterial hypertension (33%), smoking (25%), and dyslipidemia (16%) (Table 1).

**Table 1:** Baseline characteristics in 499 patients with solid or hematological neoplasms undergoing chemotherapy and/or chest radiotherapy. HF, heart failure; PAD, peripheral arterial disease; AAA, abdominal aortic aneurysm

Characteristic	Total patients n=499	Cardiotoxicity risk				
		Very high risk n (%) 220/499 (44.1)	High risk n (%) 61/499 (12.2)	Intermediate risk n (%) 70/499 (14)	Low risk n (%) 113/499 (22.6)	Very low risk n (%) 35/499 (7)
Age (years)						
Median (minimum-maximum value)	58 (18-97)	57 (18-88)	59 (24-85)	67 (24-97)	60 (18-88)	47 (19-64)
Female sex n (%)	289/499 (58)	146/289 (50.5)	41/289 (14.1)	35/289 (12.1)	67/289 (23.1)	0/289 (0)
Medical history n (%)						
Hypertension	166/499 (33)	77/166 (46.3)	19/166 (11.4)	37/166 (22.2)	33/166 (19.8)	0/166 (0)
Smoker	125/499 (25)	42/125 (33.6)	10/125 (0.8)	24/125 (19.2)	38/125 (30.4)	11/125 (8.8)
Dyslipidemia	80/499 (16)	42/80 (52.5)	7/80 (8.75)	13/80 (16.25)	18/80 (22.5)	0/80 (0)
Hypothyroidism	69/499 (13.8)	29/69 (42)	9/69 (13)	12/69 (17.3)	18/69 (26)	1/69 (1.4)
Diabetes mellitus 2	63/499 (12.6)	32/63 (50.7)	7/63 (11.1)	16/63 (25.3)	8/63 (12.6)	0/63 (0)
Chronic kidney disease	24/499 (4.8)	13/24 (54.1)	4/24 (16.6)	1/24 (4.1)	5/24 (20.8)	1/24 (4.1)
Alcoholism	22/499 (4.4)	4/22 (18.2)	1/22 (4.5)	9/22 (40.9)	7/22 (31.8)	1/22 (4.5)
Cancer	21/499 (4.2)	11/21 (52.3)	1/21 (4.7)	3/21 (14.2)	6/21 (28.5)	0/21 (0)
Coronary artery disease/PAD/AAA	20/499 (4)	9/20 (45)	1/20 (5)	9/20 (45)	1/20 (5)	0/20 (0)
Atrial fibrillation	12/499 (2.4)	8/12 (66.6)	1/12 (8.3)	1/12 (8.3)	2/12 (16.6)	0/12 (0)
Obesity	12/499 (2.4)	10/12 (83.3)	1/12 (8.3)	0/12 (0)	1/12 (8.3)	0/12 (0)
Heart failure preserved ejection fraction (>40%)	7/499 (1.4)	4/7 (57.1)	2/7 (28.5)	0/7 (0)	1/7 (14.2)	0/7 (0)
Previous radiotherapy	6/499 (1.2)	3/6 (50)	0/6 (0)	2/6 (33.3)	1/6 (16.6)	0/6 (0)
Stroke	5/499 (1)	4/5 (80)	0/5 (0)	1/5 (20)	0/5 (0)	0/5 (0)
Previous use of anthracyclines	3/499 (0.6)	2/3 (66.6)	0/3 (0)	1/3 (33.3)	0/3 (0)	0/3 (0)
HF reduced ejection fraction (<40%)	3/499 (0.6)	3/3 (100)	0/3 (0)	0/3 (0)	0/3 (0)	0/3 (0)
Valvulopathies	2/499 (0.4)	2/2 (100)	0/2 (0)	0/2 (0)	0/2 (0)	0/2 (0)

The most frequently reported type of cancer was non-Hodgkin’s lymphoma (21.6%, n=108) followed by breast cancer (19.4%, n=97), whose most common histological subtype was invasive ductal carcinoma (85.5%, n=83); colon and rectal cancer were in third place (8%, n=40).

Regarding treatment, the most commonly used chemotherapy agents were cyclophosphamide (43.8%, n=219), doxorubicin (35.2%, n=176), rituximab (23.2%, n=116), vincristine (16.2%, n=81), and paclitaxel (13.8%, n=69). Ninety-three patients (18.6%) received thoracic radiotherapy with a mean dose of 5222 cGy (SD 2072). The most common first-line chemotherapy protocols were RCHOP (n=62), AC (n=54), ABVD (n=29), CyBorD (n=25), 7+3 (n=17), and GRAALL (n=16); while the regimens associated with trastuzumab were delivered to 11 patients [AC + Docetaxel + Trastuzumab (n=2), AC + Trastuzumab (n=3), FOLFOX + Trastuzumab (n=1), Paclitaxel + Trastuzumab (n=4), Trastuzumab + Docetaxel (n=1)].

Chemotherapy-induced systolic dysfunction developed in 12% of patients receiving CyBorD (n=3/25), 11.7% with 7+3 (n=2/17), 9.6% with RCHOP (n=6/62), 6.25% with GRAALL (1/16), and 1.8% with AC (n=1/54). From the regimens associated with trastuzumab, only one patient developed systolic dysfunction due to chemotherapy (n=1/11).

Based on the Mayo clinic score, most patients presented a very high risk of cardiotoxicity (44.1%, n=220), with the very low risk being the least frequent classification (7%, n=35) (Table 2). Cardiology service was not consulted in 90% (n=449) before chemotherapy, 65% (n=325) had a baseline echocardiogram, 57% (n=284) electrocardiogram, control echocardiogram was available in 19.4% (n=97), CV metabolic profile was done in 10.6% (n =53), and cardiac biomarkers in 3% (n=15) of the population. The availability of the echocardiogram varied depending on the risk of cardiotoxicity, being reported in 84% (n=185) of the patients with a very high risk of cardiotoxicity, 80.3% (n=49) in the high risk group, 51.4% (n=36) in the intermediate risk group, 34.5% (n=39) in the low risk group, and 45.7% (n=16) in the very low risk group.

**Table 2:** Cardiotoxicity risk and cardiovascular outcomes in 499 patients with solid or hematological neoplasms undergoing chemotherapy and/or chest radiotherapy.

Cardiovascular outcome		Cardiotoxicity risk				
	Total patients n (%)	Very high risk n (%) 220/499 (44.1)	High risk n (%) 61/499 (12.2)	Intermediate risk n (%) 70/499 (14)	Low risk n (%) 113/499 (22.6)	Very low risk n (%) 35/499 (7)
Chemotherapy associated left ventricular systolic dysfunction	22 (4.4)	11/22 (50)	5/22 (22.7)	2/22 (9)	2/22 (9)	2/22 (9)
Death from any cause	80 (16)	44/80 (55)	10/80 (12.5)	7/80 (8.7)	15/80 (18.7)	4/80 (5)
Tachyarrhythmia	14 (2.8)	6/14 (42.8)	3/14 (21.4)	2/14 (14.2)	3/14 (21.4)	0/14 (0)
Cardiovascular hospital admission	7 (1.4)	5/7 (71.4)	1/7 (14.2)	0/7 (0)	1/7 (14.2)	0/7 (0)
Bradyarrhythmia	1 (0.2)	1/1 (100)	0/1 (0)	0/1 (0)	0/1 (0)	0/1 (0)
Acute myocardial infarction	4 (0.8)	2/4 (50)	0/4 (0)	0/4 (0)	1/4 (25)	1/4 (25)
Stroke	1 (0.2)	0/1 (0)	0/1 (0)	0/1 (0)	0/1 (0)	1/1 (100)
Global longitudinal strain decrease >15%	0 (0)	0 (0)	0 (0)	0 (0)	0 (0)	0 (0)

Measurement of global longitudinal strain was performed on 23.7% (n=77) of the baseline echocardiograms and 32% (n=31) of the control echocardiograms, in which was not possible to identify a >15% drop in any of the cases. Left ventricular systolic dysfunction due to chemotherapy occurred in 4.4% (n=22) of patients, all with symptoms of heart failure; this complication was observed predominantly in patients older than 50 years (91%, n=20). All-cause death was presented in 16% (n=80). The incidence of tachyarrhythmias was 2.8% (n=14), with atrial fibrillation being the most frequent (n=5), followed by atrial flutter (n=3), tachycardia atrial (n=2), and sinus tachycardia (n=2). Other outcomes are displayed in Table 2.

## Discussion

This is the first study evaluating cardiotoxicity in the adult cancer population in Colombia. The median age of our cohort was 58 years, with a high proportion of CV comorbidities, mainly arterial hypertension (33%), smoking (25%), and dyslipidemia (16%). Although our population had a very high risk of cardiotoxicity in 44% (229) of the patients, 90% were not evaluated by cardiology before the chemotherapy was started. Pre-chemotherapy echocardiogram was obtained in 65% of the population and only 19.4% underwent echocardiographic control during follow-up. Chemotherapy-related left ventricular systolic dysfunction developed in 4.4% and rhythm disturbances in 2.8%, with atrial fibrillation and atrial flutter as the most frequent arrhythmias. Patients who developed cardiotoxicity showed in general low CV risk factor burden and were predominantly affected by hematological malignancies. This analysis suggests that the cardiac complications found in these patients were the result of cancer therapy itself.

Our complication rate is similar to the reported in previous studies [16-17]. In the study by Henry et al. [16], which included 16,456 patients diagnosed with breast cancer treated with chemotherapy, 4.2% developed cardiotoxicity (defined as the presence of symptoms of heart failure). Thavendiranathan et al. in a retrospective cohort that included 18,540 women with breast cancer undergoing chemotherapy, reported a cumulative incidence of the primary outcome of 3.8% (composite of hospitalization, admission to the emergency room for heart failure, pulmonary edema or cardiomyopathy, as well as the outpatient diagnosis of chronic heart failure and CV death) [17].

In our study, the most frequent outcomes were left ventricular systolic dysfunction with heart failure symptoms (4.4%) and tachyarrhythmias (2.8%), being more prevalent in the population older than 70 years. A great proportion of patients were not followed-up by a cardiologist and this may lead to underdetection of arrhythmia disorders. Ruiz-Mori et al. [18], in a retrospective cohort of 985 patients with solid or hematological neoplasms undergoing chemotherapy, observed that arrhythmias were the most frequent adverse effect in 41.2%, followed by angina pectoris 18.7%, while heart failure only occurred in 4.9% of patients.

Thavendiranathan et al. [17] found that patients whose treatment included trastuzumab in combination with other chemotherapeutic agents without anthracyclines had a higher risk of developing cardiotoxicity, compared to patients who received another type of chemotherapy (hazard ratio: 1.76). In the case of the study conducted by Henry et al. [16], the rate of cardiotoxicity described in the trastuzumab group was 8.3%, with a median of 8 months until the event compared to 2.7% in those who did not receive it. In our study, of the patients who received this drug, only one presented systolic dysfunction by echocardiography; however, these data may be underrepresented due to the limited availability of control echocardiography and follow-up by cardiology.

In contrast to what was observed in the Mayo Clinic cohort, where breast cancer was the most frequent neoplasm (56.7%) [15], our most common cancer was non-Hodgkin's lymphoma (21.6%), followed by breast cancer (19.4%). This can be explained by a reference bias as the institution where the study was carried out is a reference center for the treatment of hematological neoplasms and the patients were recruited predominantly during hospital admissions.

Chavez-MacGregor et al. [19] conducted a retrospective study in which they evaluated the CV follow-up of 2,203 patients diagnosed with stage I-III breast cancer undergoing trastuzumab-based chemotherapy. In this study, adequate CV follow-up was defined in those patients who underwent baseline echocardiography or radionuclide ventriculography (4 months prior to the first dose) and a follow-up evaluation every 4 months while receiving therapy. Only 36% of the included patients fulfilled an adequate CV evaluation according to the defined parameters. In contrast to the above, in our study, 65% had baseline echocardiography, but only 19.2% had control echocardiography and 10% had an evaluation by cardiology. Of the total baseline echocardiograms requested (n=325), 14.7% (n=48), 33.8% (n=110), and 51.3% (n=167) were performed in 2017, 2018, and 2019, respectively, which denotes a better recognition of CV risk associated with chemotherapy as the years progressed. One of the motivations for carrying out this study, in addition to the lack of regional information, is to promote the development of programs that allow strict monitoring and prevent the development of CV events associated with cardiotoxicity in these patients.

Limitations

We recognize as limitations that this study was carried out in a single high-complexity academic center, which limits the external validity when contrasting with other clinical contexts. There is a risk of reference bias, as it included patients with more comorbidities and with an increased risk of potential for complications. Data collection was done retrospectively and was based solely on information taken from the electronic medical record.

Imaging studies were requested at the discretion of the treating physician, this aspect predisposes to selection bias. Finally, due to the low number of patients undergoing control echocardiography, comparisons between multiple echocardiographic parameters could not be effectively established.

Despite these limitations, this study provides clinical and epidemiological characteristics regarding CV outcomes that may help to develop risk stratification models for chemotherapy-induced cardiotoxicity, as well as help clinicians to better manage cardiotoxic effects while maximizing its oncological benefits.

Future studies may expand cardiotoxicity risk assessment in multicenter and population-based settings, evaluating the impact of pre-existing CVD. In addition, studies developing cardiotoxicity risk assessment tools may also be considered to identify high-risk groups and facilitate clinical decision support in cancer care.

## Conclusions

We identified in our cohort that despite a high-risk population, patients with cancer undergoing high-toxicity chemotherapy do not receive adequate prechemotherapy CV assessment. Hematological malignancies were the most common and the most common complications were heart failure (HF) and tachyarrhythmias. The development of cardio-oncology programs will allow to protocolize care, identify early potential complications, and improve patient outcomes.
